# Lost companions: a new quill mite species and its possible coextinction with the Carolina parakeet

**DOI:** 10.1017/S0031182023001373

**Published:** 2024-04

**Authors:** Maciej Skoracki, Markus Unsöld, Milena Patan, Bozena Sikora

**Affiliations:** 1 Department of Animal Morphology, Faculty of Biology, Adam Mickiewicz University in Poznań, Poznan, Poland; 2 SNSB-Bavarian State Collection of Zoology, Section Ornithology, Munich, Germany

**Keywords:** Acari, birds, ectoparasites, extinction, parrots, psittaciformes, syringophilidae

## Abstract

Investigations of the parasites associated with extinct avian species provide unique insights into the ecology and evolution of both hosts and their parasitic counterparts. In the present paper, a new quill mite species, *Peristerophila conuropsis* sp. n., belonging to the family Syringophilidae (Prostigmata: Cheyletoidea) is described from the Carolina parakeet *Conuropsis carolinensis* Linnaeus (Psittaciformes: Psittacidae). This new species was collected from museum dry skin of the Carolina parakeet, the only native representative of the Psittacidae in the United States, which was an abundant resident of the southeastern and midwestern states and has been extinct in the beginning of the 20th century. Comment on the current taxonomic state and host associations of the genus *Peristerophila* are provided. Based on the host associations and habitats occupied by *Peristerophila* and related genera on parrots, it is hypothesized with the high probability that *P. conuropsis* has been extinct along with its host.

## Graphical abstract

**Figure:**
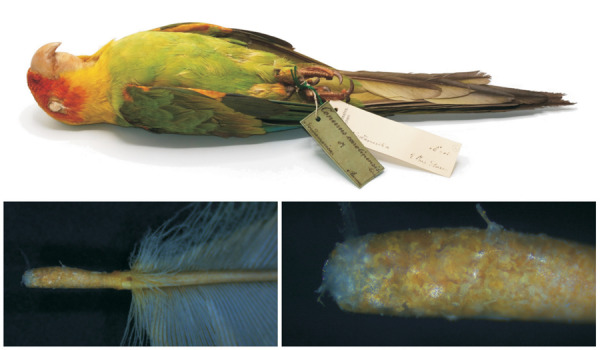

## Introduction

Quill mites of the family Syringophilidae (Prostigmata: Cheyletoidea) are specialized parasites of birds. These parasites occupy a distinct ecological niche, i.e., the interior cavities of feather quills, where they spend most of their life, feed and reproduce (Kethley, 1970, 1971; Skoracki, 2011). Among quill-dwelling mites (e.g., Apionacaridae, Ascouracaridae, Syringobiidae), this family is the most diverse taxonomic group and can be found in various microhabitats of their hosts, i.e., feathers of different types: primaries, secondaries, wing and tail coverts, and body contour feathers. Currently, approximately 400 described species of syringophilid mites in 2 subfamilies, parasitize birds from 27 out of the 44 extant avian orders (Skoracki *et al*., 2023; Zmudzinski *et al*., 2023).

Parrots (Psittaciformes) are remarkable for harbouring a highly diverse array of quill mites both in their taxonomy and morphology. Over the course of the past half-century, 45 species grouped into 8 genera and 2 subfamilies have been recorded on parrots belonging to all extant families, Cacatuidae, Psittacidae, Psittaculidae and Strigopidae (Fain *et al*., 2000; Bochkov and Perez, 2002; Bochkov and Fain, 2003; Skoracki, 2005; Skoracki and Sikora, 2008; Glowska and Laniecka, 2013; Skoracki and Hromada, 2013; Marciniak *et al*., 2019*a*, 2019*b*; Marciniak-Musial and Sikora, 2022; Marciniak-Musial *et al*., 2022, 2023). The Carolina parakeet, *Conuropsis carolinensis* Linnaeus, 1758 (Psittacidae) was examined as part of an ongoing project focusing on the collection of parasitic mites from birds deposited in the Bavarian State Collection of Zoology (Munich, Germany).

The Carolina parakeet was the only indigenous parrot species in the United States, with 2 subspecies, *C. c. carolinensis* (Linnaeus) distributed from Virginia to Florida, and *C. c. ludoviciana* (Gmelin) distributed across the Mississippi–Missouri River drainages (Clements *et al*., 2023). These colourful birds measured 30 cm in length and lived in dense flocks in the woods, primarily near rivers or swamps, where fed on seeds and fruits (Hume, 2017; Snyder and Russell, 2020). This species was regarded as an agricultural pest because flocks significantly damaged farmland. Furthermore, ‘sportsmen’ slaughtered them in large numbers for amusement; it was quite easy to kill an entire flock as the parakeets would continually return to check on their deceased flock mates, that made these birds easy targets. Additionally, hundreds were captured alive annually for the pet trade and zoological gardens. The last confirmed sighting in the wild was a flock of 13 specimens in Florida in April 1904 (Hume, 2017). In 1917, ‘*Lady Jane*’, the female of the last captive pair at the Cincinnati Zoo, died, leaving her male counterpart ‘*Incas’* as the last surviving member of the species. *Incas* died in the following year, and his body was frozen in a block of ice for shipment to the Smithsonian Institution in Washington, similar to ‘*Martha*’, the last Passenger Pigeon *Ectopistes migratorius*, who had died 4 years earlier at the same zoo. Unlike *Martha*, however, the *Incas's* body has never reached its intended destination (Fuller, 2014).

Regrettably, the Carolina parakeet eluded thorough biological research prior to its disappearance. Consequently, numerous details about its ecological niche and the exact reasons for its extinction are likely to remain uncertain or a matter of conjecture (Snyder and Russell, 2020). Currently, more than 700 skins of the Carolina parakeet are preserved in collections worldwide, serving as valuable resources for parasitological research. It is worth noting that among mites permanently associated with birds, 6 new feather mite species belonging to the 3 families (Astigmata: Pterolichidae, Psoroptoididae and Xolalgidae) have been reported from the Carolina parakeet (Mironov *et al*., 2005), whereas prostigmatan mites have never been recorded from this host.

## Materials and methods

**Figure 1.**
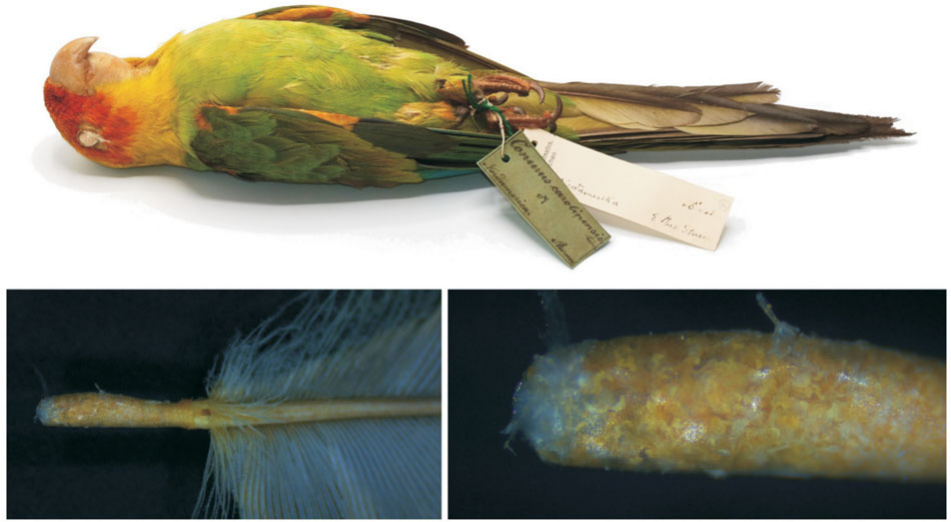
Specimen of the Carolina parakeet housed in the Bavarian State Collection of Zoology, Munich, Germany, and the infested feather quill.

Mites were collected from the lesser wing covert of the single museum skin of the Carolina parakeet housed at the Bavarian State Collection of Zoology, Munich, Germany (Fig. 1). An infested feather was carefully opened using a stereomicroscope and 2 sharp-tipped tweezers. For light microscopy, mites were initially softened in Nesbitt's solution at 40°C for approximately 48 hours and then mounted on slides in Faure's medium (Walter and Krantz, 2009).

Identification of the mite specimens and the preparation of drawings were carried out using a ZEISS Axioscope 2 (Carl-Zeiss AG, Germany) light microscope equipped with Difference-Interference-Contrast (DIC) optics and a camera lucida. All measurements are given in micrometres, with the range for paratypes provided in parentheses following the data for the holotype. The nomenclature for idiosomal chaetotaxy follows that of Grandjean (1939), as adapted for Prostigmata by Kethley (1990). The leg setation follows Grandjean (1944), and general morphological terms follow Skoracki (2011).

Specimen depositories and reference numbers are cited using the following abbreviations AMU – Adam Mickiewicz University, Department of Animal Morphology, Poznan, Poland; SNSB-ZSM – Bavarian State Collection for Zoology, Section Arthropoda Varia, Munich, Germany.

## Results

### Family Syringophilidae Lavoipierre, 1953 Subfamily Syringophilinae Lavoipierre, 1953 Genus *Peristerophila* Kethley, 1970 ***Peristerophila conuropsis* sp. n.** (Fig. 2)

**Figure 2.**
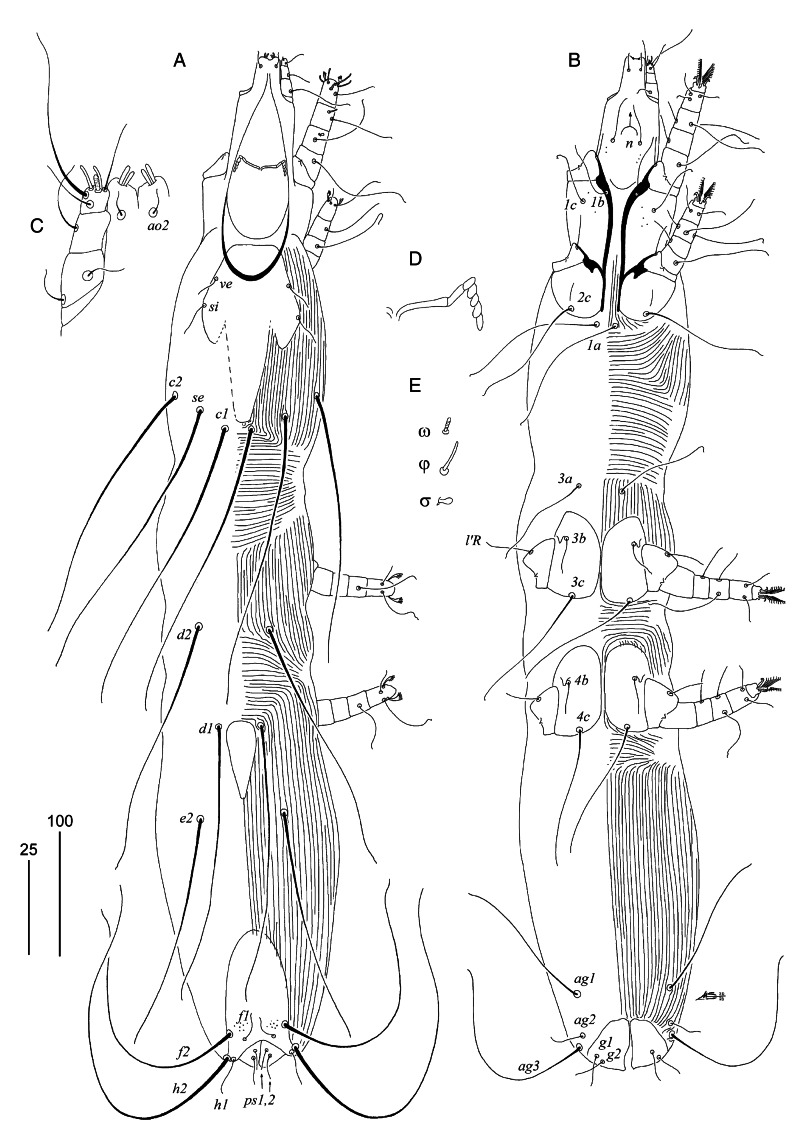
*Peristerophila conuropsis* sp. n., female. (A) dorsal view; (B) ventral view; (C) gnathosoma in ventral view; (D) peritreme; (E) solenidia of leg I. Scale bars – A, B = 100 *μ*m; C–E = 25 *μ*m.

Female, holotype. Total body length 810 (760–865 in 12 paratypes). Body view as in Figs 2A, B. Gnathosoma. Hypostomal apex with 2 pairs of large finger-like protuberances (Fig. 2C). Infracapitulum sparsely punctate. Each medial branch of peritremes with 3 chambers, each lateral branch with 4 chambers (Fig. 2D). Stylophore 195 (180–195) long; exposed portion of stylophore apunctate, 150 (145–150) long. Movable cheliceral digit 145 (145–150) long. Idiosoma. Propodonotal shield, entire, bearing bases of setae *ve* and *si*, covered with minute punctations. Bases of setae *c1* situated posterior to level of setal bases *se*; *c2* situated anterior to level of *se* or both pair of setae situated at same transverse level. Setae *ve* and *si* short (shorter than 30); length ratio of setae *ve*:*si* 1:1.3–1.7. Setae *se*, *c1* and *c2* long (longer than 200) and subequal in length. Hysteronotal shield reduced to small sclerite situated between setal bases *d1*–*d1* and *e2*–*e2*. Bases of setae *d1* situated equidistant between setal bases *d2* and *e2*. Setae *d1*, *d2* and *e2* subequal in length. Pygidial shield with rounded anterior margin, sparsely punctate near bases of setae *f1* and *f2*. Length ratio of setae *f1*:*h1*:*f2*:*h2* 1:1:7–9:12–13; *ag1*:*ag2*:*ag3* 3–5.2:1:4.5–6.2. Genital plate absent. Genital and pseudanal setae subequal in length or setae *g2* and *ps2* 1.3–1.5 times longer than *g1* and *ps1*. Coxal fields I sparsely punctate, II–IV apunctate; coxal fields III in close proximity to each other, with anterior margin not reaching bases of setae *3a*. Setae *3c* 2.7–4.2 times longer than *3b*. Cuticular striations as in Fig. 2A, B. Legs. Fan-like setae *p′* and *p″* of legs I with 5–7, II with 6–8, III and IV with 10–11 tines. Solenidia of legs I as in Fig. 2E. Femora I punctate ventrally, other podomers apunctate. Lengths of setae: *ve* 20 (15–20), *si* 30 (25–30), *se* 240 (220–240), *c1* 245 (225–245), *c2* 220 (200–225), *d1* 230 (210–225), *d2* 205 (200–205), *e2* 195 (185–195), *f1* 25 (25–35), *f2* 225 (220–225), *h1* 25 (25–30), *h2* 310 (305–320), *g1* 20 (15–20), *g2* 20 (20–30), *ps1* 20 (15–20), *ps2* 25 (20–30), *ag1* 120 (100–130), *ag2* 25 (25–35), *ag3* 155 (130–150), *tc*′*III–IV* 15 (15), *tc″III–IV* 60 (55–60), *3b* and *4b* 30 (25–35), *3c* and *4c* 110 (95–110), *l*′*RI* (15–25), *l*′*RII* (25–30), *l*′*RIII* 30 (30–40), *l*′*RIV* 25 (25–30).

Male. Not found.

**Type material.** Female holotype and paratypes: 12 females, 3 tritonymphs and 3 protonymphs from the quill of small wing covert of the Carolina parakeet *C. carolinensis* Linnaeus, 1758 (Psittaciformes: Psittacidae); North America, no other data.

**Type material deposition.** Holotype and paratypes are deposited in the SNSB-ZSM (reg. no. SNSB-ZSM A20112209), except for 5 female paratypes in the AMU (reg. no. MS 23-0621-001).

**Differential diagnosis.** *Peristerophila conuropsis* sp. n. is morphologically most similar to the *P. nestoriae* Marciniak *et al*. 2019*a*, 2019*b*, described from the New Zealand Kaka, *Nestor meridionalis* (Gmelin) (Psittaciformes: Strigopidae), in New Zealand (Marciniak *et al*., 2019*b*). In females of both species, the propodonotal shield is entire, and the hysteronotal shield is reduced to the small sclerite situated between setal bases *d1*–*d1* and *e2*–*e2*. This species differs from *P. nestoriae* by the following features: in females of *P. conuropsis*, the length of the stylophore is 180–195; bases of setae *c1* are situated posterior to the level of setal bases *se*; the hysteronotal setae *d2* and *e2* are subequal in length; the anterior margin of coxal fields III not reaching bases of setae *3a*, and the lengths of setae *e2* and *f2* are 185–195 and 220–225, respectively. In females of *P. nestoriae*, the length of the stylophore is 130–140; bases of setae *c1* and *se* are situated in the same transverse level; the hysteronotal setae *d2* are 1.2–1.5 times longer than *e2*; the anterior margin of coxal fields III reaching bases of setae *3a*, and the lengths of setae *e2* and *f2* are 116–146 and 140–155, respectively.

**Etymology.** The specific name ‘*conuropsis*’ is taken from the generic name of the host and is a noun in apposition.

## Discussion

Exploration of parasites from extinct bird species yields distinctive insights into the ecology and evolution of the hosts and their parasitic associates. Parasites often exhibit intimate associations with the biology, ecology and evolution of their hosts, rendering them a vital source of information regarding extinct species. They can provide insights into the diet, behaviour and living environment of extinct bird species. Moreover, studies on parasites and their extinct hosts can yield data regarding reciprocal adaptations and coevolutionary processes. And finally, unveiling new parasite species from extinct bird species can provide insights into the biodiversity and ecology of parasites in the past.

Until now, the order Psittaciformes has a distinctive quill mite fauna consisting of 45 species spread across 8 genera. Currently, mites from the Syringophilidae family have been identified from 82 parrot species of all extant families, Cacatuidae, Psittacidae, Psittaculidae and Strigopidae (Marciniak-Musial *et al*., 2023). As for the whole family Syringophilidae, most syringophilid species associated with parrots are restricted to a single host species (monoxenous parasites; 63% of the total quill mite fauna associated with parrots). Mite species that are associated with phylogenetically closely related host species within the same genus (stenoxenous parasites; 18%) or family (oligoxenous parasites; 17%) constitute a minority. A negligible portion of the syringophilid fauna related to parrots includes species that infest more or less unrelated host species, being polyxenous parasites (2%) (Marciniak-Musial *et al*., 2023).

One of the genera associated with parrots is the genus *Peristerophila*, to which the newly described species belongs. This genus boasts the broadest host spectrum among all known syringophilid genera. It includes 14 species and uniquely inhabits not only parrots but also hawks (Accipitriformes), falcons (Falconiformes), pigeons and doves (Columbiformes), hoopoes (Bucerotiformes) and bee-eaters (Coraciiformes) (Casto, 1976; Skoracki *et al*., 2010, 2017, 2020, 2021; Kaszewska *et al*., 2020). The *Peristerophila* fauna associated with parrots includes 3 species noted on representatives of the parrot families Psittaculidae, Psittacidae and Strigopidae. Of these 3 mite species, 2 are monoxenous and exclusively related to parrots *Peristerophila nestoriae* Marciniak *et al*. 2019*a*, 2019*b*, is associated with the New Zealand Kaka *Nestor meridionalis* (Gmelin) (Strigopidae) in New Zealand, and *Peristerophila forpi* (Bochkov and Perez, 2002) lives on with the Mexican Parrotlet *Forpus cyanopygius* (Souancé) (Psittacidae) in Mexico (Bochkov and Perez, 2002; Marciniak *et al*., 2019*b*). The third species, *Peristerophila mucuya* Casto, 1980, is currently regarded as a polyxenous parasite inhabiting several hosts from the orders Psittaciformes, i.e., the white-winged parakeet *Brotogeris versicolurus* (St. Muller) (Psittacidae) from Brazil; the gray-hooded parakeet *Psilopsiagon aymara* (d'Orbigny) (Psittacidae) from South America, and the coconut lorikeet *Trichoglossus haematodus* (Linnaeus) (Psittaculidae) from Indonesia, and also occurring on pigeons and doves (Columbiformes: Columbidae) (Bochkov and Fain, 2003; Kaszewska-Gilas, *et al*., 2021; Marciniak-Musial and Sikora, 2022), although, it is possible that this species represents a series of cryptic species which are more host-specific.

Taking into account that the mite family Syringophilidae largely comprises highly host-specific species, in most cases represented by monoxenous parasites (Skoracki, 2011; Skoracki *et al*., 2016), it is highly probable that the extinction of the Carolina parake*et al*so led to the extinction of this particular parasite species, *P. conuropsis*. It might also be suggested that this species is an oligoxenous parasite restricted to birds closely related to each other, e.g., belonging to 1 genus. However, an issue arises here because the genus *Conuropsis*, established by Linnaeus, is monotypic. An attempt could be made to find this species on its closest living relatives. The majority of researchers have proposed that *Conuropsis* is most closely related to the genus *Aratinga*, a deduction derived from shared morphological characteristics (Forshaw, 1989; Snyder, 2004). Mitochondrial DNA extracted from museum specimens of the Carolina parakeet strongly supported a sister relationship with a clade that includes *Aratinga nenday* (Vieillot), *A. solstitialis* (Linnaeus) and *A. auricapillus* (Kuhl) (Kirchman *et al*., 2012). However, according to Skoracki (2011), 2 different syringophilid species do not co-occupy the same habitat type in the plumage; thus, the presence of *Peristerophila* on hosts from the genus *Aratinga* is unlikely. This is because these birds host a different quill mite species from the genus *Neoaulobia* Fain *et al*., 2000, which occupies the same habitat type (wing coverts) being also typical for *Peristerophila*. Therefore, with a high degree of probability, it should be accepted that the parasite species *P. conuropsis* has become extinct along with its host.

## Data Availability

All the data analysed in the current paper can be made available on request to MS.
